# Recombinase-Aided Amplification Coupled with Lateral Flow Dipstick for Efficient and Accurate Detection of Porcine Parvovirus

**DOI:** 10.3390/life11080762

**Published:** 2021-07-28

**Authors:** Yihong He, Wenxian Chen, Jindai Fan, Shuangqi Fan, Hongxing Ding, Jinding Chen, Lin Yi

**Affiliations:** 1 College of Veterinary Medicine, South China Agricultural University, Guangzhou 510642, China; heyihone@stu.scau.edu.cn (Y.H.); 20183073014@stu.scau.edu.cn (W.C.); fanjindai@stu.scau.edu.cn (J.F.); shqfan@scau.edu.cn (S.F.); dinghx@scau.edu.cn (H.D.); 2 Key Laboratory of Zoonosis Prevention and Control of Guangdong Province, Guangzhou 510642, China; 3 Guangdong Laboratory for Lingnan Modern Agriculture, Guangzhou 510642, China

**Keywords:** porcine parvovirus, recombinase-aided amplification, lateral flow dipstick

## Abstract

Porcine parvovirus (PPV) infection is the primary cause of SMEDI (stillbirth; mummification; embryonic death; infertility) syndrome, which is a global burden for the swine industry. Thus, it is crucial to establish a rapid and efficient detection method against PPV infection. In the present work, we developed a recombinase-aided amplification (RAA) assay, coupled with a lateral flow dipstick (LFD), to achieve an amplification of PPV DNA at 37 °C within 15 min. The detection limits of PPV RAA-LFD assay were 10^2^ copies/μL recombinant plasmid pMD19-T-VP1, 6.38 × 10^−7^ ng/μL PPV DNA, and 10^−1^ TCID_50_/mL virus, respectively. This method was highly specific for PPV detection with no cross-reactivity for other swine pathogens. In contrast to polymerase chain reaction (PCR), the PPV RAA-LFD assay is more sensitive and cost-saving. Hence, the established PPV RAA-LFD assay provided an alternative for PPV detection, especially in resource-limited regions.

## 1. Introduction

Porcine parvovirus (PPV) is a small non-enveloped, single-stranded DNA virus belonging to the *Parvoviridae* family, the *Parvovirinae* subfamily and the Ungulate *protoparvovirus I* species [1]. It leads to a financially damaging disease known as PPV infection, characterized by SMEDI (stillbirth; mummification; embryonic death; infertility) syndrome [1,2]. Seven PPV genotypes have been identified and designated as PPV1, PPV2, PPV3, PPV4, PPV5, PPV6, and PPV7 [3]. Firstly isolated from sows [4], PPV is endemic worldwide, seriously influencing herd profitability due to the reproduction failure it causes [5], especially primiparous sows. Furthermore, it is also related to diarrhea and cutaneous lesions [6,7,8]. Thus far, no practical therapeutic interventions exist and, consequently, early detection of PPV infection is essential for PPV prevention. In light of this, there is an urgent need to develop an efficient and accurate detection system for PPV detection.

Developed as a new technique, recombinase-aided amplification (RAA) can amplify the DNA fragment over a short period of time. DNA is synthesized without thermal cycling because recombinase UvsX, DNA polymerase, and single-strand DNA binding (SSB) protein serve as alternatives. RAA recombinase can tightly bind to primer at 35–42 °C to form complexes. Primer unwinds DNA with the help of SSB protein after the complementary sequence is identified, and DNA polymerase drives the template synthesis from 3′-terminal of primers to create newly replicated DNA. These steps were consistently repeated to achieve exponential amplification [9]. RAA combined with a lateral flow dipstick (LFD) made detective results visible to the naked eye. The dual-label amplification product was obtained due to the addition of a 6-carboxy-fluorescein (FAM)-labeled probe and biotin-labeled primers. The products are binded to FAM-antibodies and detected by the interaction between biotin and anti-biotin antibodies at the test line.

Herein, we developed a rapid, convenient and accurate RAA-LFD assay for the detection of PPV, targeting the conserved gene VP1 of PPV. It will be conducive to execute a point-of-care analysis, especially in resource-limited areas, thus promising broad clinical applications [10].

## 2. Materials and Methods

### 2.1. Virus and Sample

Porcine parvovirus (PPV, GD strain), porcine circovirus2 (PCV2, YHW strain), Japanese encephalitis virus (JEV, sw/GD/2009 strain), classical swine fever virus (CSFV, Shimen strain), porcine reproductive and respiratory syndrome virus (PRRSV, GD08-2 strain), pseudorabies virus (PRV, Ea strain), and Senecavirus A (SVA, GD-ZYY02-2018 strain) were stored at our laboratory. All clinical samples were kept in our laboratory. The viral P72 plasmid of the African swine fever virus (ASFV) was kindly provided by another laboratory.

Viral DNA and RNA extraction were performed using the Viral DNA Kit (OMEGA Bio-Tek, Norcross, GA, USA) and the Viral RNA Kit (OMEGA Bio-Tek, Norcross, GA, USA), respectively, and then preserved at −80 °C for subsequent experiments. The probe and primers were synthesized by Shanghai Sangon Biotech (Shanghai, China).

### 2.2. Construction of the Standard Recombinant Plasmid pMD19-T-VP1

The primers used for standard recombinant plasmid construction are listed in Table 1. Viral DNA fragments were recovered from agarose gels after the polymerase chain reaction (PCR) had completed. The recovered, purified products were ligated into pMD19-T, then the plasmid pMD19-T-VP1 was extracted using a Plasmid Mini Kit I (OMEGA Bio-Tek, Norcross, GA, USA).

### 2.3. PCR-AGE for PPV

The PCR primers for PPV detection are listed in Table 2 [11]. A PCR was conducted with a volume of 50 µL, containing 45 µL of GreenMix (Tsingke Biotechnology, Beijing, China), 1 µL of DNA template, 2 µL of forward primer (10 µM), and 2 µL of reverse primer (10 µM), thereby yielding a 203 bp amplicon of the VP1 gene. Cycling parameters were as follows: initial denaturation (98 °C, 2 min); 30 cycles of denaturation (98 °C, 10 s), annealing (57 °C, 30 s), and extension (72 °C, 15 s); final extension (72 °C, 2 min). Products were detected on a 1% agarose gel electrophoresis (AGE).

### 2.4. RAA-AGE for PPV

Three sets of primers, targeting the conserved region of the VP1 gene, were designed according to the design principle of RAA primers (Table 3).

The RAA-AGE assay was performed in a volume of 50 µL according to the instructions of the RAA Nucleic Acid Amplification Kit (Jiangsu Qitian Gene Biotechnology, Jiangsu, China). 47.5 μL of a mixture containing 2.4 µL of forward primer (10 μM), 2.4 µL of reverse primer (10 μM), 25 µL of buffer, 2 µL of DNA template, and 15.7 µL of purified water was transferred into the reaction tubes containing lyophilized enzyme powders provided by the kit. Then, 2.5 µL of magnesium acetate (280 mM) was added to initiate the reaction after the lyophilized powders had fully dissolved. Reaction tubes were placed into the water bath at 37 °C for 15–20 min. Finally, the optimal primer pair was selected by 1.5% agarose gel electrophoresis.

### 2.5. PPV RAA-LFD Assay

The probe used in this study was design based on the optimal primer pair according to the principle of RAA. It was labeled with FAM at the 5′-end and contained a tetrahydrofuran abasic-site mimic (THF) in the middle, while the probe was modified with a C3-spacer (Polymerase extension blocking group) at the 3′-end. The reverse primer was labeled with biotin at the 5′-end.

The reaction system of RAA-LFD assay was 50 µL, which included 2.1 µL of forward primer (10 μM), 2.1 µL of reverse primer (10 μM), 0.6 µL of probe, 25 µL of buffer, 2 µL of DNA template, 15.7 µL of purified water, and 2.5 µL of magnesium acetate (280 mM). RAA products were directly tested by using a commercial lateral flow dipstick (Ustar Biotechnologies, China). In brief, 10 μL of RAA products were dropped onto a sample pad of the lateral flow dipstick, and then the sample pad was inserted downward into the microplate containing 100 μL of buffer (Ustar Biotechnologies, China). After 15–30 min, the test results were recorded. Results were interpreted as positive when two lines occurred (test and control lines) and as negative when only a control line appeared. Otherwise, the result was considered invalid.

### 2.6. Optimization of Reaction Conditions for RAA-LFD Assay

The determination of the optimal reaction temperature range was performed by incubating the RAA reaction for 20 min at varying temperatures (25 °C, 30 °C, 35 °C, 40 °C, 45 °C). Temperatures within the range defined in the previous step were further detected. The optimization of reaction time was conducted by incubating varying reaction times for 5 min, 10 min, 15 min, 20 min, 25 min, and 30 min at the optimized temperature, respectively.

### 2.7. The Specificity and Sensitivity of PPV RAA-LFD Assay

A specificity evaluation for the PPV RAA-LFD assay was conducted by detecting several swine viruses (PPV, PCV2, JEV, CSFV, PRRSV, PRV, SVA, and ASFV). The sensitivity evaluation was compared with PPV PCR-AGE assay and PPV RAA-AGE assay by detecting a ten-fold serially diluted recombinant plasmid pMD19-T-VP1 (1.0 × 10^1^ to 1.0 × 10^10^ copies/μL), PPV DNA (6.38 × 10^−9^ to 6.38 × 10^1^ ng/μL), and PPV virus with different titers (1.0 × 10^−4^ to 1.0 × 10^4^ TCID_50_/mL), respectively.

### 2.8. Evaluation of Clinical Samples

Thirty-eight suspected samples stored in our laboratory were simultaneously tested with the PPV RAA-AGE assay and the PPV PCR-AGE assay to evaluate the feasibility of the clinical application, respectively.

## 3. Results

### 3.1. Primers Screening for RAA-AGE Assay

**Figure 1:**
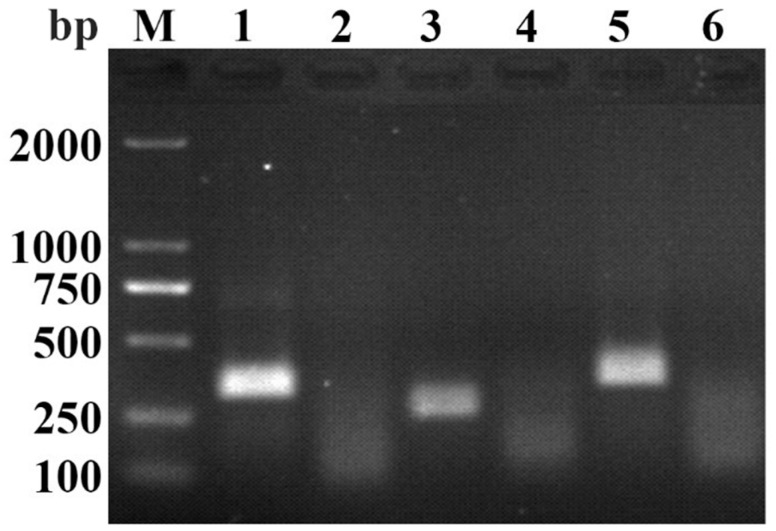
Screening of optimal primer pair using the RAA-AGE assay. M, DL2000 DNA Marker; 1, PPV-RAA-1; 2, PPV-RAA-1-negative control; 3, PPV-RAA-2; 4, PPV-RAA-2-negative control; 5, PPV-RAA-3; 6, PPV-RAA-3-negative control.

Three primer pairs were tested in the RAA-AGE assay. Results showed that the RAA-AGE assay, using a primer pair of PPV-RAA-F1 and PPV-RAA-R1, produced a brighter band compared with the other primer pairs (Figure 1). Hence, PPV-RAA-F1 and PPV-RAA-R1 were selected as the optimal primer pair. The probe was designed according to the optimal primer pair. The optimal primer pair and probe are listed in Table 4.

### 3.2. The Establishment for RAA-LFD Assay and Optimization of Reaction Conditions

**Figure 2:**
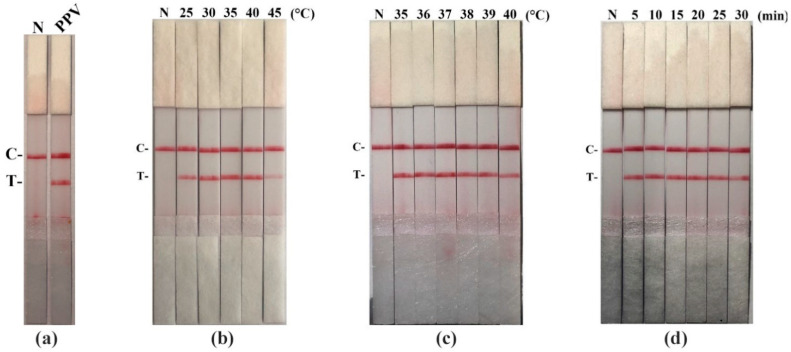
Establishment and optimization of the PPV RAA-LFD assay. (**a**) Establishment of the PPV RAA-LFD assay. N, ddH_2_O (negative control); PPV, PPV DNA template (positive control); C, control line; T, test line; (**b**) Optimization of reaction temperature. N, ddH_2_O (negative control); C, control line; T, test line; (**c**) Optimization of reaction temperature. N, ddH2O (negative control); C, control line; T, test line; (**d**) Optimization of reaction time. N, ddH_2_O (negative control); C, control line; T, test line.

Firstly, we established the PPV RAA-LFD assay (Figure 2a). Then, we figured out the optimization of reaction conditions. PPV RAA-LFD performed in various temperatures (25 °C, 30 °C, 35 °C, 40 °C, and 45 °C) at 20 min. Results revealed the optimal temperature ranged between 35 °C and 40 °C (Figure 2b). To further obtain the optimal temperature, we carried out further optimization. The result showed that test lines yielded at 36 °C, 37 °C, and 38 °C were more obvious compared to those at other temperatures (Figure 2c). 37 °C was thus chosen as the optimal temperature.

We next performed the optimization of RAA reaction time. Different incubation times (5 min, 10 min, 15 min, 20 min, 25 min, and 30 min) were tested at 37 °C. The test lines began to appear at around the time of 5 min. As the RAA incubation time was extended, test lines show no significant changes until 20 min. The test strips incubated for 25 min and 30 min show both blurrier test line and control line than others (Figure 2d). Herein, in order to obtain a stable result, we chose 15 min as the optimal reaction time.

### 3.3. The Specificity for PPV RAA-LFD Assay

**Figure 3:**
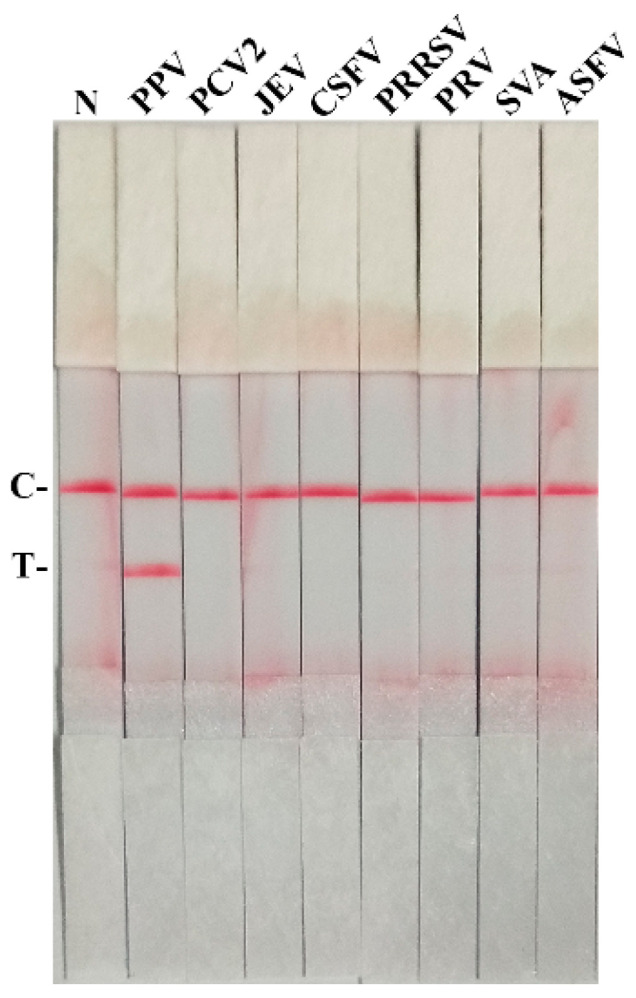
The specificity analysis of PPV RAA-LFD assay. N, ddH_2_O (negative control); PPV, porcine parvovirus; PCV2, porcine circovirus2; JEV, Japanese encephalitis virus; CSFV, classical swine fever virus; PRRSV, porcine reproductive and respiratory syndrome virus; PRV, pseudorabies virus; SVA, Senecavirus A; ASFV, African swine fever virus; C, control line; T, test line.

Different viruses (PPV, PCV2, JEV, CSFV, PRRSV, PRV, SVA, and ASFV) were detected by the PPV RAA-LFD assay. No test line was observed except the PPV template (Figure 3), indicating that the PPV RAA-LFD assay can specifically detect PPV.

### 3.4. The Sensitivity for PPV RAA-LFD Assay

**Figure 4:**
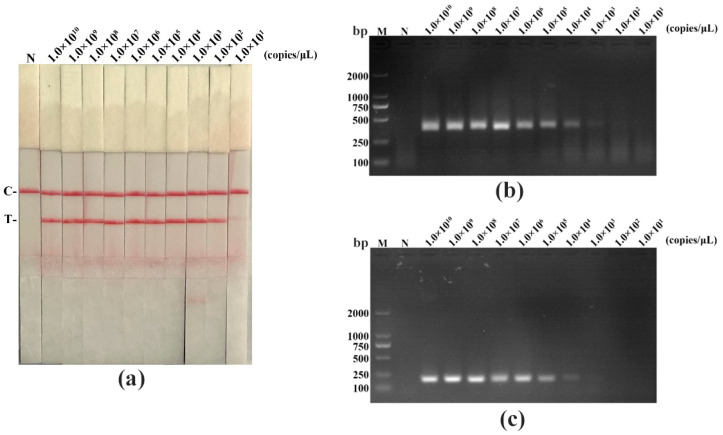
The sensitivity evaluation of the PPV RAA-LFD assay by detecting a ten-fold serially diluted recombinant plasmid pMD19-T-VP1 compared with the PPV RAA-AGE assay and the PPV PCR-AGE assay. (**a**) PPV RAA-LFD assay. N, negative control; C, control line; T, test line; (**b**) PPV RAA-AGE assay. M, DL2000 DNA Marker; N, negative control; (**c**) PPV PCR-AGE assay. M, DL2000 DNA Marker; N, negative control.

**Figure 5:**
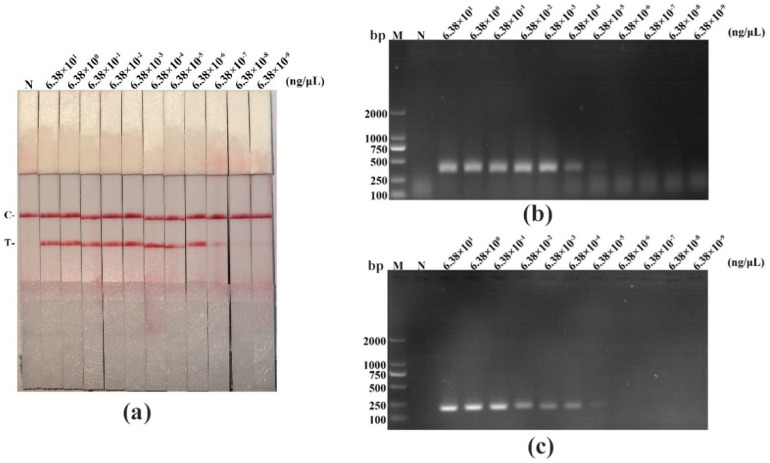
The sensitivity evaluation of the PPV RAA-LFD assay by detecting a ten-fold serially diluted PPV DNA compared with the PPV RAA-AGE assay and the PPV PCR-AGE assay. (**a**) PPV RAA-LFD assay. N, negative control; C, control line; T, test line; (**b**) PPV RAA-AGE assay. M, DL2000 DNA Marker; N, negative control; (**c**) PPV PCR-AGE assay. M, DL2000 DNA Marker; N, negative control.

**Figure 6:**
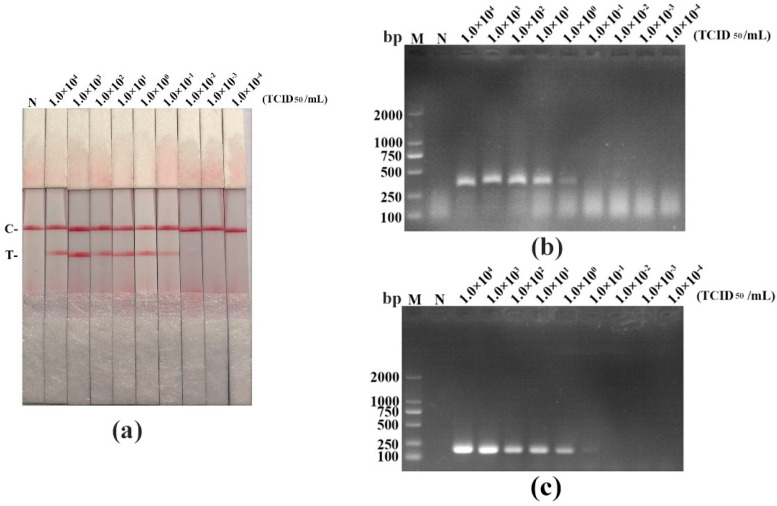
The sensitivity evaluation of the PPV RAA-LFD assay by detecting different titers of PPV compared with the RAA-AGE assay and the PCR-AGE assay. (**a**) PPV RAA-LFD assay. N, negative control; C, control line; T, test line; (**b**) PPV RAA-AGE assay. M, DL2000 DNA Marker; N, negative control; (**c**) PPV PCR-AGE assay. M, DL2000 DNA Marker; N, negative control.

We performed a sensitivity experiment to analyze the minimum detection limits of recombinant plasmid pMD19-T-VP1, PPV DNA, and virus titer in diverse detective methods (RAA-LFD, RAA-AGE, and PCR-AGE), respectively. As shown in Figure 4, Figure 5 and Figure 6, and in Table 5, RAA-AGE and PCR-AGE had a similar sensitivity; RAA-LFD was more sensitive than both RAA-AGE and PCR-AGE. In detail, RAA-LFD could detect as low as 10^2^ copies/μL of the recombinant plasmid, 6.38 × 10^−7^ ng/μL of PPV DNA, and 0.1 TCID_50_/mL of the virus, respectively. Our results indicated the PPV RAA-LFD assay was highly sensitive.

### 3.5. Clinical Samples Detection of PPV RAA-LFD Assay

**Figure 7:**
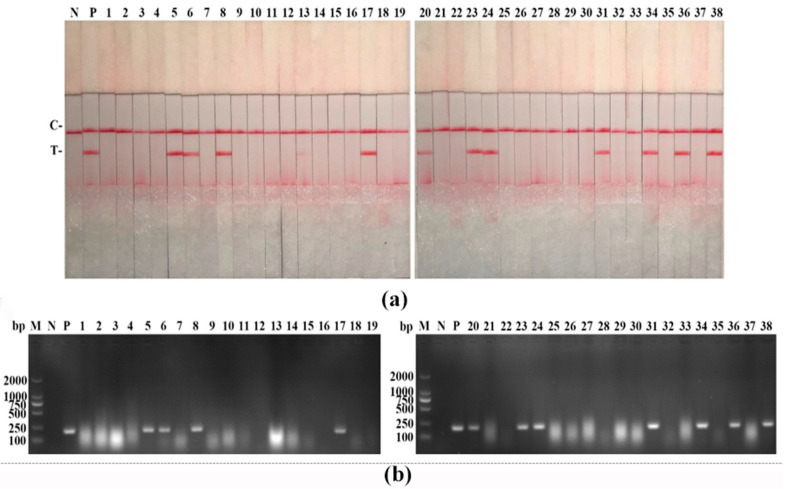
Clinical samples detection of the PPV RAA-LFD assay. (**a**) PPV RAA-LFD assay. N, negative control; P, positive control; C, control line; T, test line; (**b**) PPV PCR assay. M, DL2000 DNA Marker; N, negative control; P, positive control.

To evaluate the performance of the PPV RAA-LFD assay, we detected 38 suspected samples simultaneously using the PPV RAA-LFD assay and the PPV PCR-AGE assay. As shown in Figure 7, both of these assays detected 11 positive samples for PPV, demonstrating 100% identical results. In Table 6, 11 positive samples and 27 negative samples were identified, showing about 28.95% of the positive rate in both PPV RAA-LFD and PPV PCR-AGE. These two assays showed the same sensitivity and specificity in clinical samples.

## 4. Discussion

PPV not only causes reproductive disorder in sows but also relates to many diseases in pigs such as porcine postweaning multisystemic wasting syndrome (PMWS) and non-suppurative myocarditis [12,13]. Notably, there are no specialized therapeutic interventions for PPV infection at present. Moreover, PPV infection is a vaccine-preventable disease, but virus shedding may still occur when pigs are infected by heterologous PPV strain despite vaccination in herds [14]. Sows and neonatal piglets infected with PPV do not usually present clinical signs except reproduction disorder, which increases the difficulty for PPV diagnosis [15,16].

While the isolation and identification of pathogens are reliable diagnostic methods, they are both time-consuming and laborious [17]. Widely applied serological diagnosis methods are usually unable to differentiate between infected animals and vaccinated animals and thus have several drawbacks, for instance low specificity [18,19]. In addition, molecular detection methods, such as PCR, real-time PCR, and loop-mediated isothermal amplification (LAMP), are commonly used. PCR and real-time PCR are sensitive and specific methods but costly to perform because the thermal cycling instrumentation they require is expensive [20,21]. LAMP is an isothermal amplification method [22]. However, primer design for LAMP is complicated, and the loop primers are prone to non-specific binding, resulting in false positives [23]. Consequently, it is crucial to develop a rapid and accurate detection method to monitor PPV infection.

RAA is a novel isothermal amplification technology developed in recent years, whose principle is similar to that of recombinase polymerase amplification (RPA). The main difference between RPA and RAA is that RPA recombinase is obtained from T4 UvsX, whereas RAA recombinase is obtained from *E. coli* [9]. Similar to RPA, RAA also has some advantages compared with traditional molecular detection methods, such as PCR. Firstly, RAA can quickly and efficiently amplify DNA sequences at a constant temperature, and results can be observed by the naked eye in combination with LFD. Secondly, instead of a thermal cycler, only an inexpensive water bath is required to perform the RAA assay. Additionally, the device and reagent costs for RAA are lower than those of PCR. Briefly, RAA possesses multiple advantages of low-cost, convenient handling, low-reaction temperature, and fast-reaction speed in comparison with general molecular methods.

The RAA products can be detected via multiple methods, such as AGE, LFD, and real-time fluorescence. Nowadays, the applications of RAA in the detection of viruses, bacteria, and parasites are being widely explored. For example, Zheng et al. [24] established an RT-RAA-LFD assay for the rapid diagnosis and endemic monitoring of Coronavirus Disease-2019. Zhang et al. [25] developed a real-time fluorescence RAA detection to monitor foodborne *salmonella* contamination. Furthermore, Zhao et al. [26] established a reliable method for *Schistosoma japonicum* detection using an RAA assay. RAA, as it develops, is gradually showing its potential. In our study, we established a PPV RAA-LFD assay. A PPV RAA-LFD can rapidly amplify PPV DNA sequences without cross-reaction with other viruses and is also more sensitive than a PCR assay. Interestingly, for the sensitivity of the PPV RAA-LFD assay, we discovered a phenomenon: in the sensitivity evaluation analyzed by the spiked samples, the sensitivity of the PPV RAA-LFD was higher than those observed from the PPV PCR-AGE. However, in the evaluation of clinical samples, both assays showed the exact same sensitivity and specificity. The possible reason for this is that the viral load in clinical samples is high enough to be detected by both methods, while the viral load in spiked samples is strictly controlled. Overall, the established PPV RAA-LFD assay is free of reliance on sophisticated instruments and technicians, providing a valuable tool for resource-poor settings. In addition, the portability of the PPV RAA-LFD assay contributes greatly to the point-of-care analysis. Though the PPV RAA-LFD assay has the strengths of efficiency, specificity, and sensitivity, its drawbacks still exist. (1) High-frequency attempts, adjustment, and validation are required to obtain the optimal RAA primers using traditional primer-designed software due to the lack of specialized software for the design of RAA primers; (2) DNA extraction is required before detection. Thus, the established assay could be improved. Previous research has reported an equipment-free method for DNA/RNA extraction within 30 seconds, which could enhance the diagnostic efficiency in combination with isothermal amplification technology [27]. Our next challenge is to simplify the steps of DNA extraction and increase the number of clinical samples for PPV detection to further evaluate the generality and clinical value of our method.

## 5. Conclusions

In conclusion, the established PPV RAA-LFD assay is a cost-effective and highly sensitive detection method. PPV DNA sequences can be exponentially amplified at 37 °C within 15 min. The amplified RAA products, coupled with LFD, can be interpreted by the naked eye. Overall, we provide an accurate and efficient tool for PPV clinical diagnosis, especially in resource-limited areas.

## Figures and Tables

**Table 1:** Primers used for amplification of PPV VP1 gene fragment.

Primers	Sequence (5′-3′)	Length (bp)	Gene
PPV-F1	CACGCATCAAGACTCATACATC	1226	VP1
PPV-R1	TCTGTATCAAGTTCTTTATCCCAT		

Abbreviations: PPV = porcine parvovirus; F = forward primer; R = reverse primer.

**Table 2:** Primers for PCR assay.

Primers	Sequence (5′-3′)	Length (bp)
PPV-F2	CACAGAAGCAACAGCAATTAGG	203
PPV-R2	CTAGCTCTTGTGAAGATGTGG	

Abbreviations: PPV = porcine parvovirus; F = forward primer; R = reverse primer.

**Table 3:** Selection of primers for RAA-AGE assay.

RAA Assay	Primers	Sequence (5′-3′)	**Length (bp)**
PPV-RAA-1	PPV-RAA-F1	ACACTGGACAATCACAACAAATAACAGACTCA	340
	PPV-RAA-R1	CCTACCTGAGCTGGCCTAATTGCTGTTGCTTC	
PPV-RAA-2	PPV-RAA-F2	TACAGATATTACCTATCATGCACCAGAAAC	274
	PPV-RAA-R2	CTGTGGTAGGTTCAGTTAGTAGTTTTGGAG	
PPV-RAA-3	PPV-RAA-F3	CATACATCTAAATATGCCAGAACACGAAAC	354
	PPV-RAA-R3	GGTGTGTATGGAAGTGTGTTATTGGTGTCT	

Abbreviations: PPV = porcine parvovirus; RAA = recombinase-aided amplification; F = forward primer; R = reverse primer.

**Table 4:** Optimal primer pair and probe for RAA assay.

Primers/Probe	Sequence (5′-3′)
PPV-RAA-F1	ACACTGGACAATCACAACAAATAACAGACTCA
PPV-RAA-R1	Biotin-CCTACCTGAGCTGGCCTAATTGCTGTTGCTTC
PPV-Probe	FAM-ACAGATCTCTAGGACTGCCTCCAAAACTAC-THF-AACTGAACCTACCAC-C3 spacer

Abbreviations: PPV = porcine parvovirus; RAA = recombinase-aided amplification; F = forward primer; R = reverse primer; FAM = 6-carboxy-fluorescein; THF = tetrahydrofuran abasic-site mimic.

**Table 5:** The detection limits of the RAA-LFD assay, the RAA-AGE assay, and the PCR-AGE assay by detecting a ten-fold serially diluted recombinant plasmid pMD19-T-VP1, PPV DNA, and virus with different titers, respectively.

Template	PPV RAA-LFD	PPV RAA-AGE	PPV PCR-AGE
Recombinant plasmid (copies/μL)	10^2^	10^4^	10^4^
DNA (ng/μL)	6.38 × 10^−7^	6.38 × 10^−4^	6.38 × 10^−4^
Virus titer (TCID_50_/mL)	10^−1^	1	1

Abbreviations: PPV = porcine parvovirus; RAA = recombinase-aided amplification; LFD = lateral flow dipstick; AGE = agarose gel electrophoresis; PCR = polymerase chain reaction; TCID_50_ = 50% tissue culture infective dose.

**Table 6:** Detection results of PPV RAA-LFD assay and PPV PCR-AGE assay by detecting 38 suspected samples, simultaneously.

Method	PPV RAA-LFD		PPV PCR-AGE	
Results	Positive	Negative	Positive	Negative
	11	27	11	27
Total samples	38		38	
Positive rate	28.95%		28.95%	

Abbreviations: PPV = porcine parvovirus; RAA = recombinase-aided amplification; LFD = lateral flow dipstick; PCR = polymerase chain reaction; AGE = agarose gel electrophoresis.
